# The inhibitory effect of Yam polysaccharides on acrylamide‐induced programmed cell death in RAW 264.7 cells

**DOI:** 10.1002/fsn3.3076

**Published:** 2022-09-27

**Authors:** Jing Wang, Ying Han, Man Wang, He Li, Yujiao Sun, Xuefeng Chen

**Affiliations:** ^1^ School of Food and Biological Engineering Shaanxi University of Science & Technology Xi'An China; ^2^ Nanyuan Hospital Beijing China

**Keywords:** acrylamide, apoptosis, autophagy, P2X7, Pyroptosis, Yam polysaccharides

## Abstract

Acrylamide has been well known for its neurotoxicity, genotoxicity, carcinogenicity, etc. Recently, the immunotoxicity of acrylamide has been reported by different research groups, although the underlying mechanisms of acrylamide endangering immune systems have not been fully elucidated. In this study, mouse monocyte–macrophage cells model was used to clarify the toxic mechanism of acrylamide and the inhibitory effect of Yam polysaccharides (YPS) on acrylamide‐induced damage. We found that acrylamide induced RAW 264.7 cell death in a time‐ and concentration‐dependent manner. After acrylamide (2.0, 3.0, 4.0 mmol/L) treatment for 24 h, cell apoptosis, autophagy, and pyroptosis were observed. However, the levels of autophagy and pyroptosis decreased at a high concentration of acrylamide (4.0 mmol/L). Acrylamide upregulated P2X7 expression, but the P2X7 level was not showing a monotone increasing trend. When the P2X7 antagonist was applied, the effect of acrylamide on autophagy and pyroptosis was weakened. Additionally, acrylamide triggered the occurrence of oxidative stress and a decreased nitric oxide (NO) level. However, reactive oxygen species (ROS) generation, the decrease of heme oxygenase‐1 (HO‐1) expression, and the increase of inducible nitric oxide synthase (iNOS) expression were reversed by the inhibition of P2X7. Yam polysaccharides (50.0 μg/ml) significantly inhibited acrylamide‐induced oxidative stress and cell death (including apoptosis, autophagy, and pyroptosis). Yam polysaccharides also effectively reversed the increase of iNOS expression induced by acrylamide. However, Yam polysaccharides promoted the expression of P2X7 rather than prohibit it. These results indicated that acrylamide caused RAW 264.7 cell death due to pro‐apoptosis as well as excessive autophagy and pyroptosis. Apoptosis might be more predominant than autophagy and pyroptosis under a higher concentration of acrylamide (4.0 mmol/L). P2X7‐stimulated oxidative stress was responsible for acrylamide‐induced programmed cell death (PCD), but P2X7 showed limited regulatory effect on apoptosis. Yam polysaccharides with antioxidant activity inhibited acrylamide‐induced cell death (apoptosis, autophagy, and pyroptosis), but exerted limited effect on the acrylamide‐induced P2X7 expression. These findings would offer an insight into elucidating the immunotoxic mechanism of acrylamide and the potential approaches to control its toxicity.

## Graphical abstract

Acrylamide triggered macrophage cell apoptosis and autophagy and pyroptosis. ;P2X7 was of great significance in acrylamide‐induced cell death, but was mainly responsible for the regulation of autophagy and pyroptosis. Acrylamide stimulated oxidative stress via P2X7 in macrophage cells. Yam polysaccharides can inhibit AA‐induced cell apoptosis, autophagy and pyroptosis.

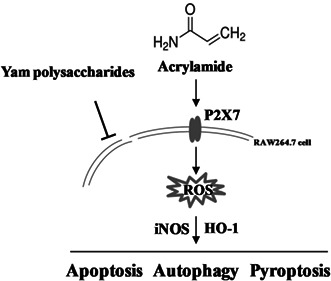

## 1 INTRODUCTION

**FIGURE 1:**
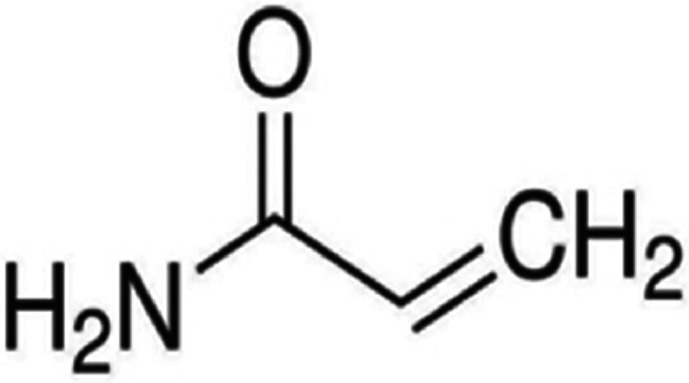
The chemical structure of acrylamide

Acrylamide (AA, 2‐propenamide, C_3_H_5_NO) (Figure 1) is formed in thermally processed foods when their reducing sugars and protein containing asparagine (amino acid) are heated at a temperature higher than 120°C and a lower moisture condition (Deribew & Woldegiorgis, 2021; Pundir et al., 2019). Acrylamide has been frequently detected in baked, fried, and roasted foods, such as potato chips, bread, coffee, biscuits, deep‐fried dough and cakes, fried nuts, and cereals (Deribew & Woldegiorgis, 2021; Pundir et al., 2019). As an unsaturated amide with high reactivity and water solubility, acrylamide has also long been used in various branches of industry for water treatment, oil exploitation, production of polyacrylamides, paper, and adhesives (Mojska et al., 2016).

As acrylamide pollution is very common in daily life, the general population is potentially exposed to acrylamide from acrylamide‐polluted water, air, and commodities, as well as dietary food while the occupational population continuously gets exposed to acrylamide in the process of synthesis and use of acrylamide. Acrylamide can enter the body through ingestion, inhalation, and skin contact, and then it is absorbed and distributed into several vital organs, causing serious harm (Matoso et al., 2019). Acrylamide can also enter the fetus through the blood of pregnant women, seriously endangering the growth and development of the fetus (Kadawathagedara et al., 2016). Because of the frequent exposure to acrylamide, its health hazard has raised worldwide attention.

Acrylamide has been well known as a neurotoxin and a probable carcinogen to humans. It is also responsible for causing the reproductive toxicity (Aras et al., 2017) and cardiac developmental toxicity (Huang et al., 2018). Moreover, the experimental animal study has recently revealed that the intake of acrylamide‐containing food in adult zebrafish (Komoike et al., 2020) caused the appearance of a melanomacrophage center and increase of major inflammatory cytokines, indicating splenic damages. Acrylamide‐treated female BALB/c mice had a significant reduction of natural killer (NK) cells and an increase of T helper cells (Th cells) (Fang et al., 2014). Based on these *in vivo* findings, acrylamide intake may cause adverse health effects on the immune system, thus the immunotoxicity of acrylamide is worth urgent study. Since acrylamide exposure has been proved to be capable of weakening the immune systems *in vivo*, continued study on the toxic mechanism is therefore of interest. However, because of the complexity of the immune system, the toxic mechanism of acrylamide has not been fully revealed. As an important part of the immune system, immune cells play an important role in the immune process. Macrophages, a kind of antigen‐presenting cells, have the phagocytic ability and play an important role in the process of immune regulation, anti‐infection and antitumor, etc. (Zhang et al., 2017). However, there are few studies on the toxicity of acrylamide to macrophages.

Programmed cell death (PCD) refers to an orderly and active way of cell death under certain circumstances or stimulations. PCD is controlled by apoptosis, pyroptosis, autophagy, etc. These types of cell death pathways share common stimuli and triggers, but each type also has its unique features and different pathways. An increasing of evidence underlined the critical role of PCD in the pathogenesis of stress and injury. Acrylamide was previously found to induce neuronal apoptosis (Li et al., 2006). Recently, some studies have shown that acrylamide could also lead to autophagy of neural cells (Xia et al., 2020) and oocytes (Aldawood et al., 2020). However, the effect of acrylamide on the programmed death of immune cells such as macrophages, and its specific mechanism are still unclear.

P2X7 (purinergic receptor P2X7) expressed in a variety of immune cells plays an important role in regulating immune response (Neves et al., 2011). P2X7 becomes activated when pathological conditions like hypoxia or cell destruction occur (Markwardt, 2021). Activated P2X7 increases membrane permeability and makes large molecules and various cations pass through, and intracellular substances flow out, resulting in a series of cell changes, such as cell lysis or apoptosis (Olivier et al., 2017; Zhu et al., 2014). Therefore, investigations of the P2X7 receptor may lead to revealing the potential targets of toxic substances. Others also reported that the P2X7 receptor can regulate the inflammatory response of macrophages. For example, activation of P2X7 strongly induced cell death in rat peritoneal macrophages (Zhu et al., 2014). The presence of functional P2X7 receptors was also confirmed in human monocyte‐derived macrophages (Vargas‐Martínez et al., 2020). However, the role of P2X7 receptors in acrylamide‐induced macrophage injury is still unclear.

Many studies have confirmed that functional polysaccharides have excellent functions in regulating immunity, lowering blood pressure, blood lipid, blood sugar, etc. There has been a rapid progress in understanding the effect of polysaccharides on immune system, especially such an effect on immune macrophages. It has been reported that nonstarch polysaccharides from Chinese Yam (Yam polysaccharides, YPS) could improve the function of RAW 264. 7 macrophages (Li et al., 2017). In previous studies, we have found that Chinese Yam polysaccharides effectively increased macrophage viability. However, it is still unknown whether Yam polysaccharides can effectively protect macrophages from acrylamide‐induced injury.

In the present study, mouse monocyte–macrophage cells were used to analyze the toxicity of acrylamide and to elucidate the death mechanisms regarding P2X7. In addition, the protective effect of Yam polysaccharides on acrylamide‐induced damage was also evaluated.

## 2 MATERIALS AND METHODS

### 2.1 Chemicals and reagents

Acrylamide was obtained from Amresco (USA). Dulbecco's modified Eagle's medium (DMEM) was provided by Gibco (Invitrogen Corporation, USA). Fetal bovine serum (FBS) was from Zeta Life (USA). MTT (3‐[4,5‐Dimethylthiazol‐2‐yl]‐2,5‐diphenyltetrazolium bromide) and dimethyl sulfoxide (DMSO) were purchased from Sigma‐Aldrich (USA). DAPI (4′,6‐diamidino‐2‐phenylindole) was from the Beyotime Institute of Biotechnology (Jiangsu, China). Assay kits for ROS, caspase‐1, tumor necrosis factor‐α (TNF‐α), lactate dehydrogenase (LDH), and bichinconinic acid (BCA) protein were purchased from Nanjing Jiancheng Bioengineering Institute (Jiangsu, China). The P2X7 receptor antagonist (A‐438079 hydrochloride) was from TargetMol. Yam polysaccharides (UV >90%) was from Wuhan JONK Biotechnology Co., Ltd. (Hube, China). The products of the antibodies, such as mouse monoclonal antibody against Beclin‐1(sc‐48341), MAP LC3α/β (sc‐398822), P2X7 (sc‐514962), iNOS (sc‐7271), GAPDH (glyceraldehyde 3‐phosphate dehydrogenase) (sc‐365062), and goat antimouse immunoglobulin G (IgG)–horseradish peroxidase (sc‐2005), were obtained from Santa Cruz Biotechnology (USA). The cleaved caspase‐3 (bsm‐33199 M) was from Bioss Antibodies and the cleaved poly ADP‐ribose protein (PARP) (ab32064) was obtained from Abcam (cambridge, UK). The β‐actin antibody (AF5003) and horseradish peroxidase (HRP)‐labeled goat antirabbit IgG (A0208) were obtained from Beyotime Institute of Biotechnology (Jiangsu, China). All other reagents were commercial products of the highest available purity grade.

### 2.2 Cell culture

RAW 264.7 cells (mouse macrophages) were provided by the National Collection of Authenticated Cell Cultures (Shanghai, China). Cells were cultured in DMEM supplemented with FBS (10%) and penicillin–streptomycin (1%) at 37°C and in an atmosphere of 5% CO_2_.

### 2.3 Cell viability assay

The MTT assay was applied to evaluate the effect of acrylamide on cell viability. Cells were seeded in 96‐well plates at a density of 8 × 10^3^/well, incubated overnight, and treated with acrylamide, A 438079 (cells were pretreated with it for 30 min) or YPS (cpretreated with for 4 h). DMEM was employed in a control group. After 12 or 24 h, MTT (0.5 mg/ml) was added to each well and incubated for 4 h. The formazan crystals formed by live cells were extracted with DMSO and the absorbance (490 nm) was recorded by a spectrophotometer (MK3, Thermo Fisher Scientific). Cell viability was calculated as per the following equation:

$$\text{The cell viability}={\mathrm{OD}}_{\text{Test Group}}/{\mathrm{OD}}_{\text{Control Group}}\times 100\%.$$

### 2.4 Phagocytosis assay

Neutral red solution was used to determine the phagocytosis of RAW 264.7 cells. Cells were seeded into a 96‐well plate (8 × 10^3^ cells per well). After 12 h, the acrylamide (2.0, 3.0, 4.0, and 5.0 mmol/L) was added to wells and incubation continued for 24 h. DMEM was employed in a control group. At the end of the culture, each well was added 100 μl of neutral red solution (0.1%, W/V) and then incubated for 10 min. After discarding the supernatant and washing the cells with PBS buffer, cell lysate (glacial acetic acid and ethanol at the ratio of 1:1, 100 μl/well) was added and placed at room temperature for 1 h. Finally, the absorbance was measured at 540 nm. The phagocytosis index was calculated by the following equation.

$$\text{Phagocytosis index}={\mathrm{OD}}_{\text{Test Group}}/{\mathrm{OD}}_{\text{Control Group}}\times 100\%.$$

### 2.5 Detection of ROS (reactive oxygen species)

The ROS production was measured with DCFH‐DA (2',7'‐ddichlorodihydrofluorescein diacetate). DCFH‐DA can produce a specific reaction with hydrogen peroxide, during which DCFH can be oxidized by hydrogen peroxide to fluorescent DCFH. After being treated with acrylamide and A438079 (P2X7 antagonist), DCFH‐DA (10 μmol/L) was added and incubated at 37°C for 30 min. Then the cells were observed and photographed under a fluorescence microscope (Olympus, IX71) or measured by a microplate reader (Varioskan Flash, Thermo Scientific).

### 2.6 Nitrite determination

Nitric oxide (NO) production was determined by measuring the content of nitrite (a stable end‐product of NO) based on the Griess reaction. Cells were seeded onto 96‐well plates and left to adhere overnight. Cells were then treated with concentrations of acrylamide for 24 h. The culture medium was mixed with an equal volume of Griess reagent (1% sulfanilamide in 5% phosphoric acid and 0.1% N‐[1‐naphthyl] ethylenediamine dihydrochloride in distilled water) and incubated at room temperature for 10 min. The absorbance was measured at 540 nm by a spectrophotometer (MK3, Thermo Fisher Scientific). A standard nitrite curve generated with sodium nitrite (in the range of 0–100 μmol/L) was applied for the quantification of NO content.

### 2.7 Caspase‐1, TNF‐α, LDH assay

Cells were seeded onto 96‐well plates and left to adhere overnight and treated with either acrylamide, A438079, or YPS. After 24 h, the levels of caspase‐1, TNF‐α, and LDH in the supernatant of the media were assessed according to the kit's instructions.

### 2.8 Western blotting analyses

Cells were treated with acrylamide, A438079, or YPS. The cell lysates were collected for western blot analysis, and their total protein concentration was measured by the BCA Protein Kit. Sodium dodecyl sulfate‐polyacrylaminde gel electrophoresis (SDS‐PAGE) was used to separate the samples of protein. Then the proteins were transferred onto polyvinylidene difluoride (PVDF) membranes (Millipore). After that, the membranes were blocked with 5% of skim milk, washed 3 times with Tris‐buffered saline with Tween 20 (TBST), and incubated with the primary antibodies (1:1000) at 4°C overnight, followed by incubation at 25°C for 2 h with secondary antibody (1:2000). The bands were visualized with a chemiluminescent substrate (Enhanced chemiluminescence (ECL), Engreen Biosystem, Beijing, China) and exposed by a Molecular Imager ChemiDoc XRS System (Bio‐Rad, Shanghai, China).

### 2.9 Statistical analysis

All the data were representative of at least three independent experiments. Results were presented as mean ± SD (standard deviation) and analyzed with SPSS 19.0 software. For comparisons of multiple samples to the control group, one‐way ANOVA (analysis of variance) was used. The value of *p* < .05 was considered statistically significant index.

## 3 RESULTS

### 3.1 Acrylamide inhibited cell growth in a time‐and concentration‐dependent manner

**FIGURE 2:**
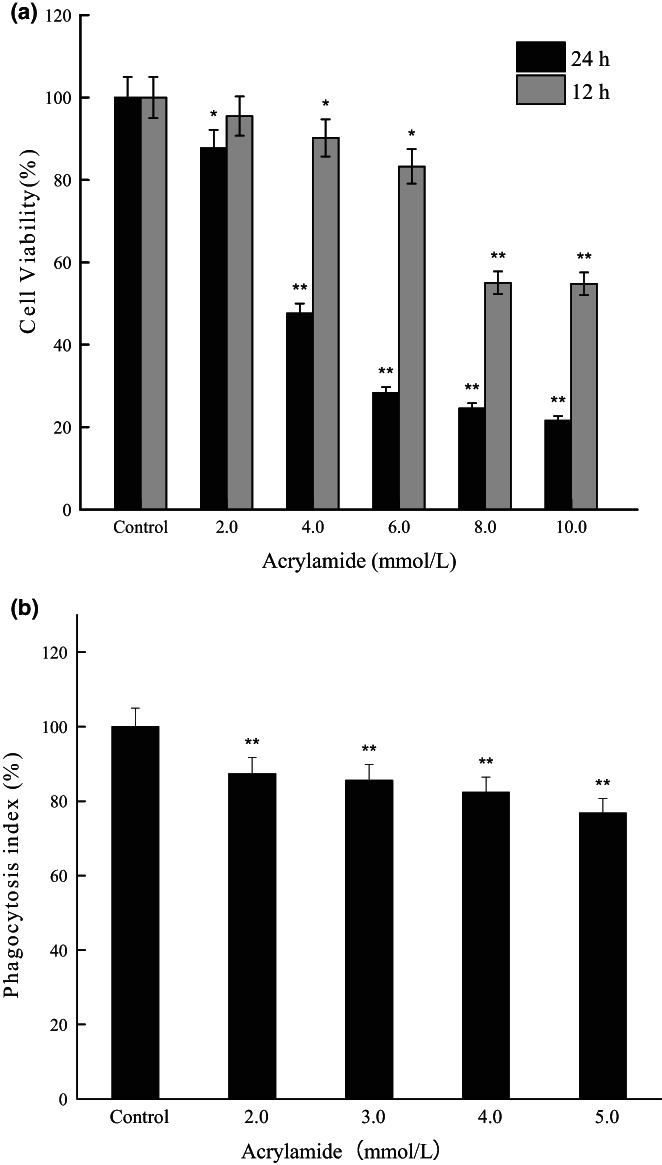
Effects of acrylamide on cell viability. (a) Cell viability was evaluated by the MTT (3‐[4,5‐Dimethylthiazol‐2‐yl]‐2,5‐diphenyltetrazolium bromide) assay after being treated with various concentrations of acrylamide for 12 and 24 h. (b) Effects of acrylamide on cell phagocytosis. Cells were treated with different concentrations of acrylamide for 24 h, and the phagocytic activity was assayed using the neutral red solution. Values were expressed as means±SD (standard deviation) (*n* = 5). *p < .05 and **p < .01 vs. control

We first evaluated the antiproliferation effect of acrylamide on RAW 264.7 cells. The results showed that acrylamide exhibited a significant time‐ and concentration‐dependent killing effect against RAW 264.7 cells, as indicated in Figure 2a. Compared with the untreated group (100.0 ± 5.0%), exposure to acrylamide at different concentrations (from 4.0 to 10.0 mmol/L) greatly inhibited cell proliferation. However, treatment for 12 h with the lower concentration (2.0 mmol/L) showed no difference (95.5 ± 4.8%). Significant inhibition was observed at the concentration of 4.0 mmol/L for 12 h (90.2 ± 4.5%) or 2.0 mmol/L for 24 h (87.7 ± 4.4%). The number of cells remarkably decreased to less than 30% after exposure to more than 6.0 mmol/L of acrylamide for 24 h. The IC_50_ value of acrylamide in RAW 264.7 cell was 4.9 mmol/L (24 h). The results suggested that acrylamide was effective in inhibiting RAW 264.7 cell growth.

### 3.2 Acrylamide inhibited cell phagocytic activity

Macrophage activation, one of the most important events in the immune response, is characterized by the prominent feature of increased phagocytosis. As shown in Figure 2b, RAW 264.7 cells in the control group had relatively high phagocytic activity (100.0 ± 5.0%). At the concentration range of 2.0–5.0 mmol/L, the acrylamide groups could steadily inhibit the activity of phagocytosis in a concentration‐dependent manner. The phagocytic activity of the cells which were exposed to 4.0 mmol/L of acrylamide for 24 h decreased to about 82.4% of the control, and the phagocytic activity with 5.0 mmol/L of acrylamide was about 76.8% of the control. These results showed that acrylamide treatment could effectively inhibit cell phagocytosis.

### 3.3 Acrylamide‐induced cell apoptosis, autophagy, and pyroptosis

**FIGURE 3:**
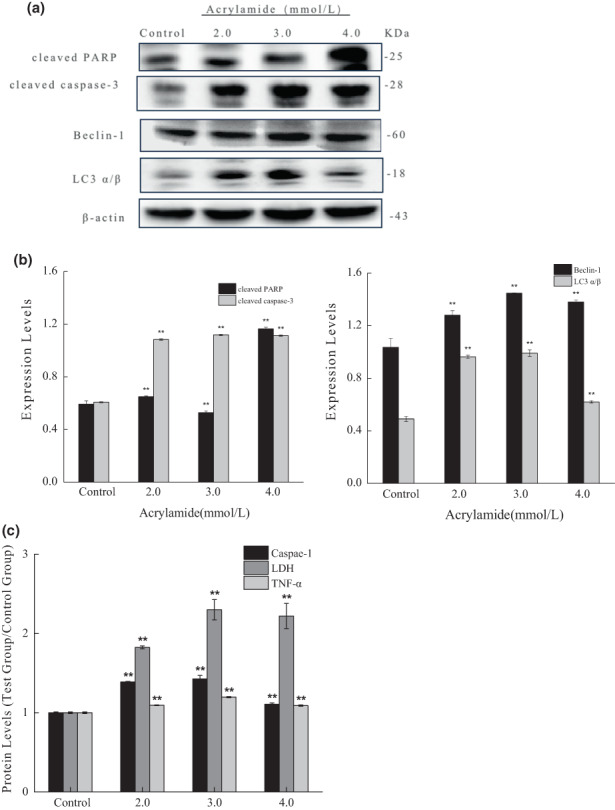
Acrylamide‐induced cell apoptosis, autophagy, and pyroptosis. The expressions of cleaved caspase‐3 and cleaved poly ADP‐ribose protein (PARP) (a), as well as Beclin‐1 and LC3 (b), were detected by western blot after cells were treated with different concentrations of acrylamide for 24 h. (c) Effects of acrylamide on caspase‐1, lactate dehydrogenase (LDH), and tumor necrosis factor‐α (TNF‐α) release were detected by an appropriate test kit. The results were presented as mean ± SD (standard deviation) (*n* = 3). *p < .05 and **p < .01 vs. control.

Apoptosis is a programmed “self‐killing,” which activates the evolutionarily conserved intracellular pathways to inhibit cell growth and proliferation (Sloviter, 2002). To identify whether acrylamide inhibits cell growth in a pro‐apoptotic way, hallmarks of apoptosis were analyzed after acrylamide treatment. Caspase‐3 is a major apoptosis mediator to cleave the poly ADP‐ribose protein (PARP) during apoptosis (Saha et al., 2018; Sloviter, 2002). As shown in Figure 3a.b, there was an obvious increase in cleaved caspase‐3 after the addition of acrylamide for 24 h. Meanwhile, a concentration‐dependent production of cleaved PARP was detected in a manner coinciding with the changes in cleaved caspase‐3 levels. These results demonstrated that acrylamide induced apoptosis in RAW 264.7 cells in a concentration‐dependent manner.

Autophagy, a catabolic process, is responsible for the degradation and recycling of cytosolic materials to allow cell survival and maintenance of cellular homeostasis (Saha et al., 2018). We continued to investigate whether acrylamide could induce autophagy in RAW 264.7 cells. The results in Figure 3a,b showed that expressions of both Beclin‐1 and LC3 (autophagy‐related proteins, light chain 3) (autophagy markers) were elevated after acrylamide treatment. However, the protein levels of Beclin‐1 and LC3 did not increase in a concentration‐dependent manner and peaked at the concentration of 3.0 mmol/L. Taken together, the results suggested that acrylamide with lower concentrations stimulated excessive cell autophagy but exerted a slight effect on autophagy at higher concentrations.

Pyroptosis, more recently, is known as inflammatory cell death. The initiating event in pyroptosis is the activation of inflammasomes, multiprotein complexes that activate caspase‐1. Pyroptotic feature eventually leads to cellular swelling, membrane rupture, and the release of inflammatory factors including LDH and TNF‐α. The results in Figure 3c showed that caspase‐1 was significantly activated after acrylamide treatment, and the release of LDH and TNF‐α increased. These results indicated that acrylamide stimulated cell pyroptosis.

### 3.4 Acrylamide‐induced cell death by P2X7 regulation

**FIGURE 4:**
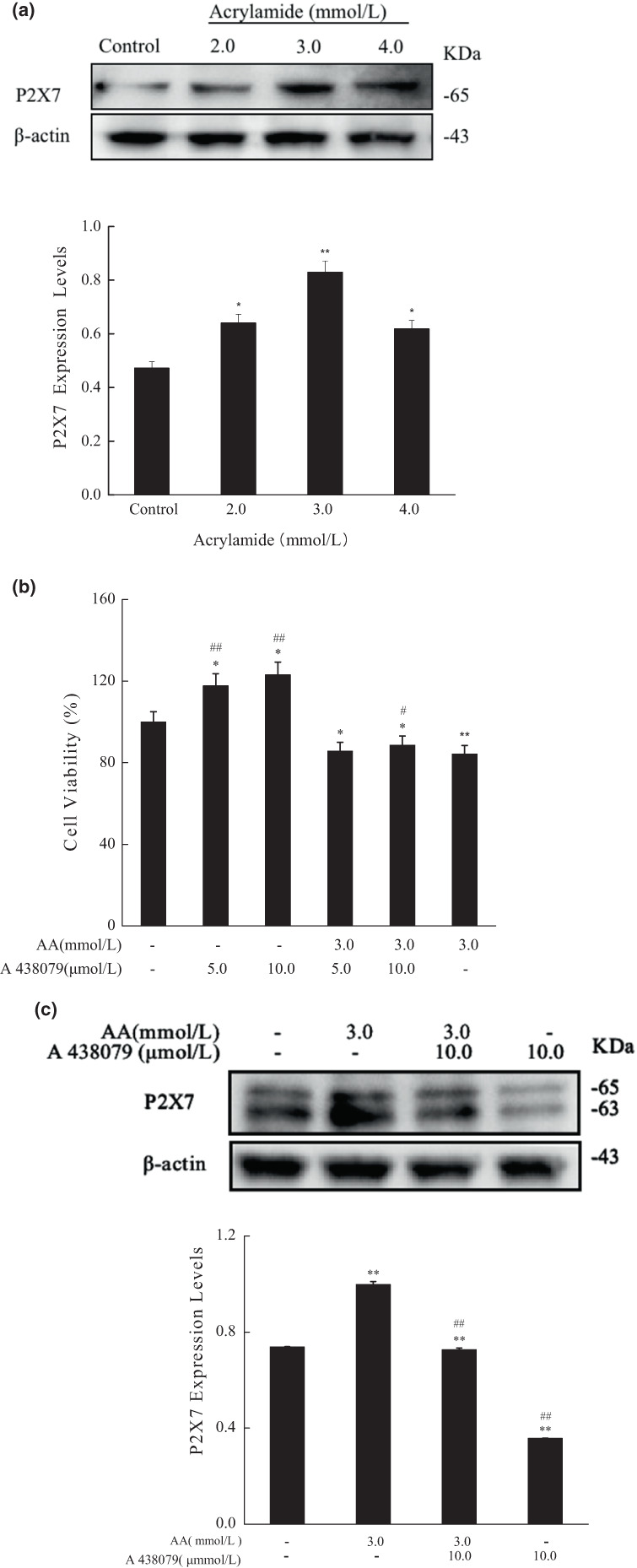
Acrylamide influenced the protein level of P2X7. (a) The protein level of P2X7 was determined by western blot after cells were treated with different concentrations of acrylamide for 24 h. (b) Cell viability was measured by the MTT (3‐[4,5‐Dimethylthiazol‐2‐yl]‐2,5‐diphenyltetrazolium bromide) assay after being treated with A 438079 for 30 min. (c) The protein level of P2X7 was determined by western blot after cells were cotreated with A438079 (10.0 μmol/L) and acrylamide (3.0 mmol/L). The results were presented as mean ± SD (standard deviation) (*n* = 3). *p < .05 and **p < .01 vs. Control, #*p* < .05 and ##*p* < .01 vs. Acrylamide (3.0 mmol/L).

P2X7 receptor, one of the subgroups of the P2X family, is a ligand‐gated ion channel. P2X7 is mainly expressed in cell types involving innate and adaptive immunity and is highly correlated with inflammation and immunity. It participates in inflammatory and nociceptive reactions of the organism to various pathological events (Chen et al., 2018; Neves et al., 2011; Souza et al., 2017). As demonstrated in Figure 4a, the expression of P2X7 was upregulated after acrylamide treatment. However, the protein level was found in an optimum responding range, but a monotone increasing trend was not observed. It reached its maximum at 3.0 mmol/L of acrylamide. Compared to the acrylamide‐alone group, the cell viability was slightly restored in the cotreated group (cotreated with 10.0 μmol/L of P2X7 antagonist [A 438079]) (Figure 4b). The cotreatment of the P2X7 antagonist also inhibited the upregulation of P2X7 protein stimulated by acrylamide (Figure 4c).

**FIGURE 5:**
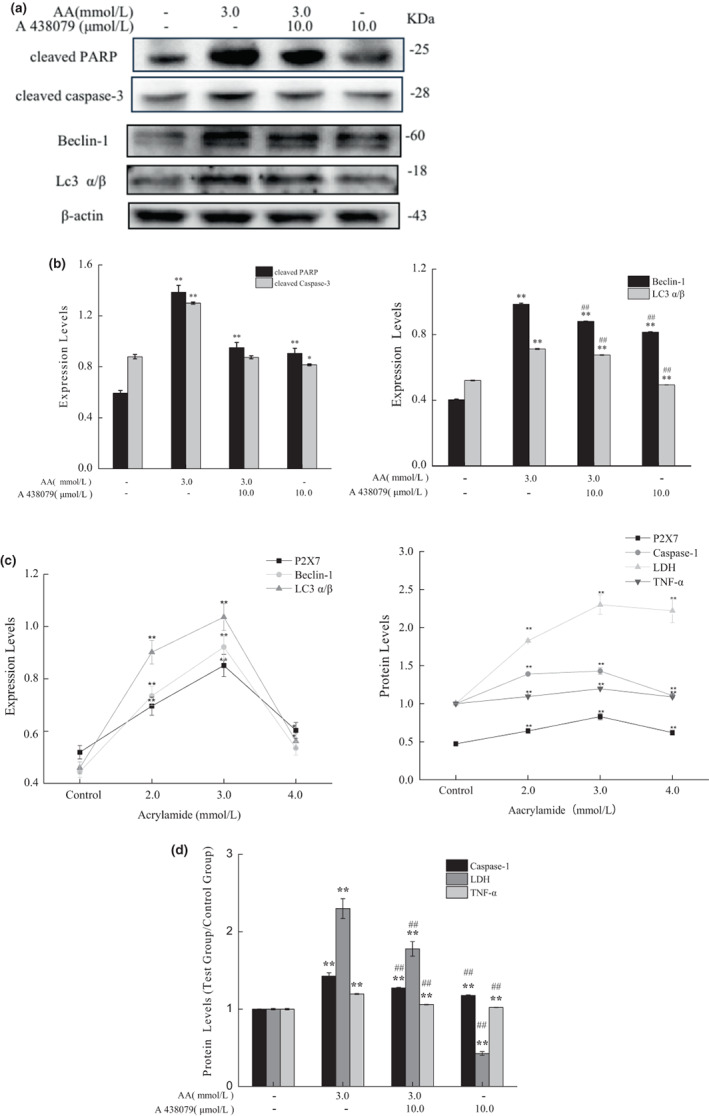
Acrylamide‐induced cell death mediated by P2X7. (a) The protein levels of cleaved PARP (poly ADP‐ribose protein), cleaved caspase‐3, Beclin‐1, and LC3 were estimated by western blot after cells were treated with A438079 (10.0 μmol/L) and acrylamide (3.0 mmol/L), the internal reference (β‐actin) was the same as Figure 4c. The quantitative results of expression intensity (b) are shown. (c) The changes in expressions of autophagic markers, pyroptotic markers, and P2X7. (d) The content of caspase‐1, lactate dehydrogenase (LDH), and tumor necrosis factor‐α (TNF‐α) in cells was assessed after treatment with A438079 (10.0 μmol/L) and acrylamide (3.0 mmol/L). The results were presented as mean ± SD (standard deviation) (*n* = 3). *p < .05 and **p < .01 vs. Control, #*p* < .05 and ##*p* < .01 vs. Acrylamide (3.0 mmol/L).

Furthermore, the P2X7 antagonist remarkably inhibited the upregulation of Beclin‐1 and LC3 induced by acrylamide, and prevented the release of LDH and TNF‐α and caspase‐1(Figure 5d). However, the production of cleaved caspase‐3 and cleaved PARP affected by acrylamide was not restored by cotreatment with P2X7 antagonist (Figure 5a,b). We also found that the alteration trend of P2X7 expression was as similar as that of autophagy markers (Beclin‐1 and LC3) and pyroptotic markers (LDH, TNF‐α, and caspase‐1), as indicated in Figure 5c. These results showed that P2X7 was vital in acrylamide‐induced cell death, but P2X7 was more likely to mediate the progress of autophagy and pyroptosis.

### 3.5 Acrylamide‐induced oxidative stress by P2X7 regulation

**FIGURE 6:**
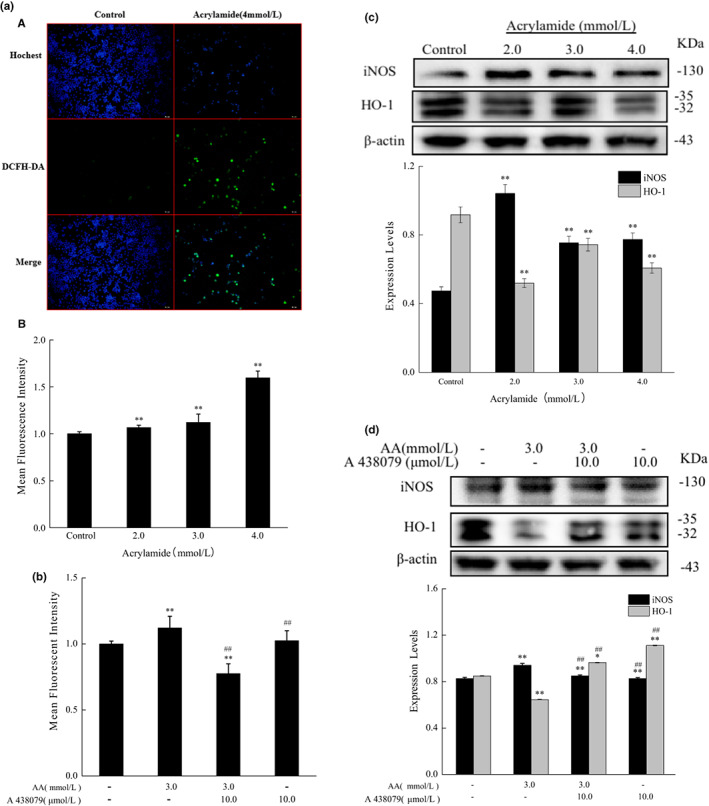
Acrylamide induced oxidative stress via P2X7 regulation in RAW 264.7 cells. (a) a) Cells were exposed to acrylamide (4.0 mmol/L) for 30 min followed by incubation with DCFH‐DA (2',7'‐Dichlorodihydrofluorescein diacetate) at 37°C for 30 min, and then were observed by a fluorescence microscope (scale bar =50 μm). b) Quantitation of reactive oxygen species (ROS) was determined by using a Varioskan flash microplate reader after cells were treated with various concentrations of acrylamide for 24 h. (b) The level of ROS was measured by a Varioskan flash microplate reader after cells were treated with A438079 (10.0 μmol/L) and acrylamide (4.0 mmol/L). (c) The protein levels of inducible nitric oxide synthase (iNOS) and heme oxygenase‐1 (HO‐1) were determined by western blot after cells were incubated with different concentrations of acrylamide for 24 h. (d) The expression of iNOS and HO‐1 was determined by western blot after cells were incubated with A 438079(10.0 μmol/L) and acrylamide (3.0 mmol/L), the internal reference (β‐actin) was the same as Figure 4c. The results were presented as mean ± SD (standard deviation) (*n* = 3). *p < .05 and **p < .01 vs. Control, #*p* < .05 and ##*p* < .01 vs. Acrylamide (3.0 mmol/L).

Intracellular ROS are considered important mediators in transduction pathways. They also play a dangerous role in inducing cell death (Bartosz, 2009). The results showed that ROS generation was significantly triggered when cells were treated with acrylamide (Figure 6a), whereas it was inhibited after the cells were cotreated with a P2X7 antagonist (Figure 6b). HO‐1(heme oxygenase‐1) is highly inducible by various stresses including pro‐inflammatory cytokines, hydrogen peroxide, toxin, and ultraviolet (UV) irradiation (Drummond et al., 2019). In this work, it was shown that HO‐1 expression was downregulated by acrylamide in a concentration‐dependent manner, which was consistent with the changes of apoptotic hallmarks (Figure 6C). Furthermore, the level of HO‐1, compared to the acrylamide‐treated group, also was upregulated when cells were cotreated with P2X7 antagonist (Figure 6D).

### 3.6 Acrylamide inhibited the production of NO but promoted the expression of iNOS

**FIGURE 7:**
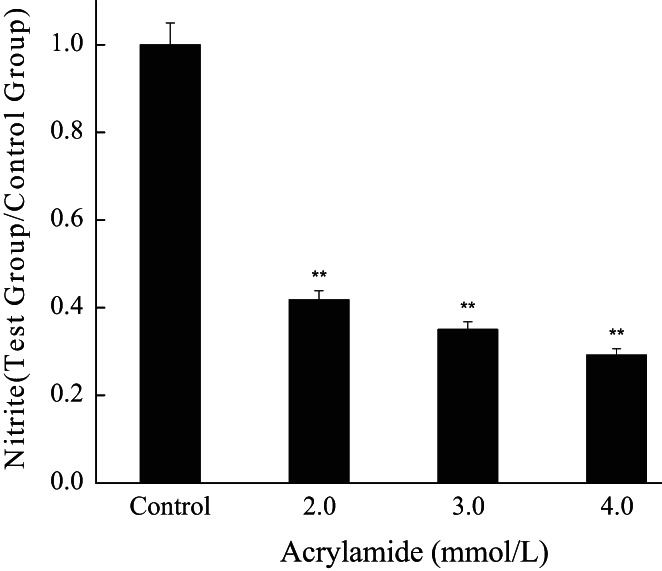
The influence of acrylamide on nitric oxide (NO) production. Cells were treated with different concentrations of acrylamide for 24 h, and then the level of NO was analyzed by colorimetric assay. *p < .05 and **p < .01 vs. control, #*p* < .05

Nitric oxide (NO) acts as a cellular signaling molecule and immune mediator in phagocytes (Zhu et al., 2015). As shown in Figure 7a, the production of NO was remarkably diminished in a concentration‐dependent manner in acrylamide‐treated cells.

iNOS (inducible nitric oxide synthase), an inducible member of the nitric oxide synthase (NOS) family, also plays a crucial role in immune and nonimmune cells during stress conditions (Anavi & Tirosh, 2020). The level of iNOS expression showed an increase in the acrylamide treatment group, but the change of iNOS expression did not show in a concentration‐dependent manner (Figure 6c). However, P2X7 did somehow inhibit the stimulation of iNOS as shown in Figure 6d. These findings indicated that oxidative stress played an important role in acrylamide‐stimulated cell death.

### 3.7 Yam polysaccharides inhibited acrylamide‐induced cell apoptosis, autophagy, and pyroptosis

**FIGURE 8:**
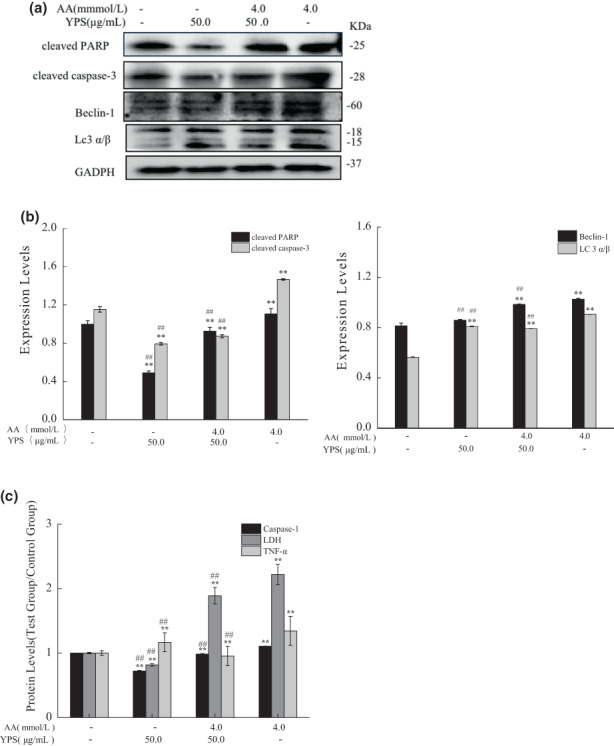
Acrylamide‐induced cell death was inhibited by Yam polysaccharides (YPS). Cells were treated with YPS (50.0 μg/ml) for 4 h followed by acrylamide (4.0 mmol/L) treatment. The expressions of cleaved caspase‐3, cleaved PARP (poly ADP‐ribose protein), Beclin‐1, and LC3 were detected by western blot(a) and quantitative results of expression intensity (b) are shown. (c)The contents of caspase‐1, lactate dehydrogenase (LDH), and tumor necrosis factor‐α (TNF‐α) were determined with an assay kit. The results were presented as mean ± SD (standard deviation) (*n* = 3). *p < .05 and **p < .01 vs. Control, #*p* < .05 and ##*p* < .01 vs. Acrylamide (4.0 mmol/L).

As illustrated in Figure 8a,b, compared with the acrylamide‐damaged group, cleaved PARP and cleave caspase‐3 in the YPS intervention group significantly decreased. However, compared with the control group, cleaved PARP slightly changed, and the level of cleaved caspase‐3 decreased to some extent. These findings indicated that Yam polysaccharides protected cells from acrylamide‐induced apoptosis.

The results in Figure 8a,b suggested that after cotreatment with YPS and acrylamide, the protein expression of Beclin‐1 and LC 3 decreased, compared with the acrylamide alone group. There was no significant change in Beclin‐1 although a slightly increased expression of LC 3 was detected in the YPS‐alone treatment group, compared with the control group. Thus, Yam polysaccharides had an inhibitory effect on acrylamide‐induced autophagy.

As shown in Figure 8c, compared with the control group, the secretion of both caspase‐1 and LDH decreased, and TNF‐α increased in the YPS‐alone treatment group. The levels of caspase‐1, LDH, and TNF‐α in the cotreatment group were significantly lower than those in the acrylamide‐alone treatment group. These results showed that Yam polysaccharides inhibited cell pyroptosis induced by acrylamide.

### 3.8 The effect of Yam polysaccharides on acrylamide‐induced P2X7 expression

**FIGURE 9:**
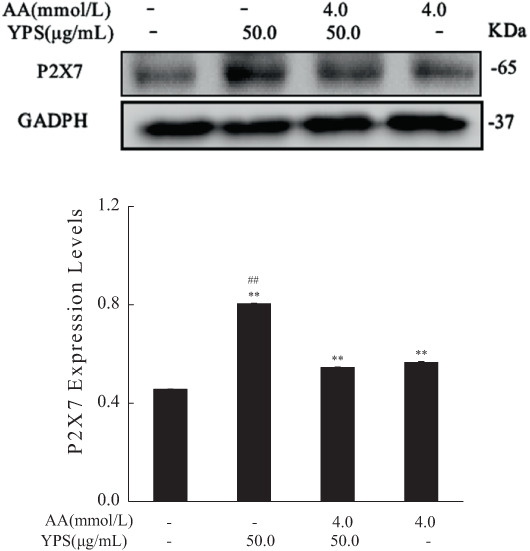
The effect of Yam polysaccharides (YPS) on the protein level of P2X7. The protein level of P2X7 was analyzed by western blot after cells were cotreated with YPS (50.0 μg/ml) and acrylamide (4.0 mmol/L), the internal reference (GAPDH (glyceraldehyde 3‐phosphate dehydrogenase)) was the same as Figure 8a. The results were presented as mean ± SD (standard deviation) (*n* = 3). *p < .05 and **p < .01 vs. Control, #*p* < .05 and ##*p* < .01 vs. Acrylamide (4.0 mmol/L).

The expression of P2X7 was upregulated after acrylamide treatment, whereas the protein level was not downregulated when cotreated with YPS (Figure 9). Interestingly, the protein level of P2X7 somehow rose by YPS alone, compared with the control group. It is assumed that Yam polysaccharides could not effectively inhibit the expression of P2X7 protein induced by acrylamide.

### 3.9 The effect of Yam polysaccharides on acrylamide‐induced protein expressions of HO‐1 and iNOS

**FIGURE 10:**
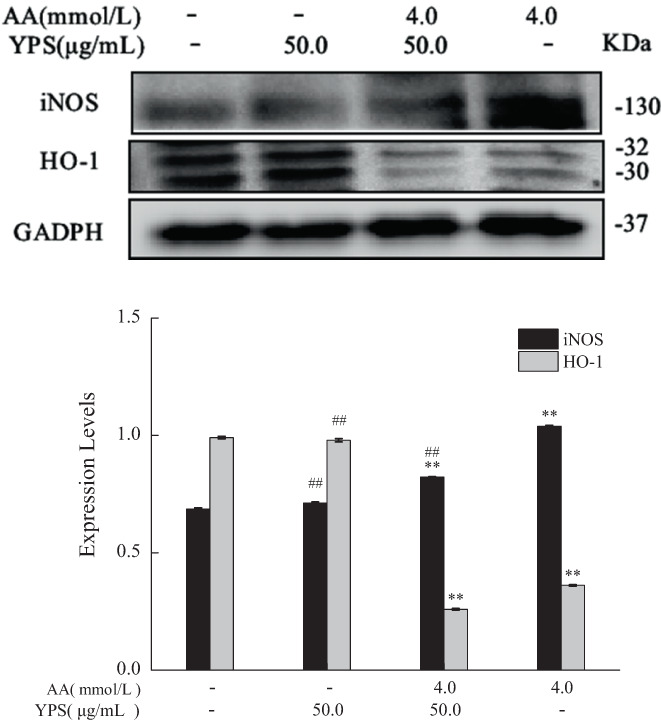
The effect of Yam polysaccharides (YPS) on the protein levels of inducible nitric oxide species (iNOS) and heme oxygenase‐1 (HO‐1) The protein levels of iNOS and HO‐1 were estimated by western blot after cells were cotreated with YPS (50.0 μg/ml) and acrylamide (4.0 mmol/L), the internal reference (GAPDH (glyceraldehyde 3‐phosphate dehydrogenase)) was the same as Figure 8a. The results were presented as mean ± SD (standard deviation) (*n* = 3). *p < .05 and **p < .01 vs. Control, #*p* < .05 and ##*p* < .01 vs. Acrylamide (4.0 mmol/L).

As demonstrated in Figure 10, there was no significant difference in the expression levels of iNOS and HO‐1 in the YPS‐alone treatment group, compared to the control group. The expression level of iNOS in the acrylamide‐alone treatment group increased, whereas it significantly decreased when cotreated with YPS and acrylamide. Unexpectedly, the level of HO‐1 expression inhibited by acrylamide was not restored by YPS and acrylamide cotreatment, and it even showed a slight decrease in YPS and acrylamide cotreatment group. These results showed that Yam polysaccharides inhibited the oxidative stress induced by acrylamide by blocking the expression of iNOS rather than restoring the level of HO‐1.

## 4 DISCUSSION

Acrylamide exhibited a severe toxic effect on macrophage cells. Both cell proliferation and phagocytosis are inhibited by acrylamide. We go on to try to elucidate its death‐promoting mechanism. It has already been reported that apoptosis was observed in in vivo models after exposure to acrylamide. In this study, it was shown that acrylamide also triggered macrophage cell apoptosis. Is apoptosis the only killing way of acrylamide? Documented evidence has suggested that apoptosis as type I programmed death interacts with autophagy (type II programmed death) to determine cell fate (Xie et al., 2020; Yuan et al., 2021). Autophagy is a process in which cells maintain the balance of content generation and degradation. Either excessive autophagy or insufficient autophagy leads to a large number of cell death and loss of cell function (Saha et al., 2018). A large number of apoptosis are mediated by autophagy (Bekker et al., 2021; Jin et al., 2020). What's more, autophagic toxicity has been addressed for a variety of environmental pollutants, such as formaldehyde (Han et al., 2015), perfluorooctane sulfonate (PFOS) (Liu et al., 2020), and cadmium (Li et al., 2020). However, there are few studies that report the autophagy of immune cells induced by acrylamide. So, we further analyzed the effect of acrylamide on autophagy in macrophage cells. We found that acrylamide also stimulated macrophage cell autophagy. However, the level of autophagy did not steadily increase with the increasing concentration of acrylamide. The autophagy level of cells treated with a high concentration (4.0 mmol/L) of acrylamide was lower than that of cells treated with a low concentration (3.0 mmol/L). It seems that acrylamide‐induced autophagy at high concentration was not obvious and serious, and this was not consistent with the fact that more cell death was detected in a higher concentration of acrylamide treatment.

Pyroptosis is a kind of cell death that is always associated with inflammation induction (Ketelut‐Carneiro & Fitzgerald, 2021). The wide occurrence of this PCD process in various cell types suggests its importance in various disorders. Pyroptosis may occur at the cellular level upon detection of cell stress. Pyroptotic cells then release many pro‐inflammatory cytokines and other mediators. Although some caspases have been discovered to induce pyroptosis, caspase‐1 has initially been known as an important inducer in the process of pyroptosis. Pyroptosis activation may lead to the upregulation of multiple pro‐inflammatory cytokines, including TNF‐α, resulting in tissue/cell damage (Wei et al., 2021). TNF‐α has been reported to help trigger pyroptosis as well as amplify the pro‐inflammatory response. Acrylamide has been reported to induce inflammation in Kupffer cells and Sprague‐Dawley (SD) rats liver by increasing cleaved caspase‐1 expression as well as TNF‐α secretion (Nan et al., 2021). In this study, the elevated level of caspase‐1, TNF‐α, and LDH suggested the involvement of cell pyroptosis in acrylamide‐damaged process. Interestingly, the changing trend of pyroptosis was consistent with that of autophagy.

Unlike autophagy and pyroptosis, apoptosis was induced in a concentration‐dependent manner. Therefore, apoptosis, autophagy, and pyroptosis were all responsible for acrylamide‐induced cell death, but apoptotic death was likely more dominant than autophagic and pyroptotic death after high concentration treatment. This is consistent with those previous findings that different types of cell death can share the same stimulus, but the threshold of induction may differ (Su et al., 2018; Wu et al., 2020).

The results showed that the protein level of P2X7 was upregulated by acrylamide, but the highest level was observed at the concentration which also detected the most severe autophagy and pyroptosis. There were some previous findings indicating that P2X7 can regulate apoptosis of macrophage cell (Placido et al., 2006), retinal cell (Zhang et al., 2018), and mice myeloblasts (Paredes‐Gamero et al., 2007). We further discovered that P2X7 antagonist cotreatment with acrylamide significantly inhibited the overexpression of P2X7 protein and the activation of autophagy and pyroptosis in acrylamide‐treated cells. And the alteration trend of P2X7 expression agreed well with the changes of autophagic and pyroptotic markers. However, these did not occur for acrylamide‐induced apoptosis. Our findings indicated that acrylamide‐induced cell autophagy and pyroptosis were mediated by P2X7, but the underlying interactions need further exploration.

As an ion channel, P2X7 has been reported to be involved in mediating cellular stress responses, such as ROS generation (Zhang et al., 2018). ROS from normal metabolism and xenobiotic exposure exert beneficial or harmful effects on cells and tissues by their relative amount. An excess of ROS disturbs the intracellular homeostasis of the redox system and destroys cell functions via oxidative damage (Bartosz, 2009). HO‐1, beneficial to treatment in several pathological conditions, generally plays a cytoprotective part when cells suffer from oxidative stress. Lack of HO‐1 was associated with the risk of oxidative injury and with sensitiveness to the cytotoxicity (Drummond et al., 2019). In this study, an increased accumulation of ROS and a decreased expression of HO‐1 were observed in a concentration‐dependent manner after acrylamide treatment. Moreover, inhibition of P2X7 worked effectively in prohibiting ROS generation and restoring HO‐1 levels, suggesting the participation of P2X7 in stimulating oxidative stress. The increase of cell apoptosis was accompanied by the increasing oxidation when a higher acrylamide concentration was applied. Interestingly, cells were more likely to maintain a relatively low level of autophagy and pyroptosis under a higher concentration of acrylamide than cells treated with a lower concentration of acrylamide. We hypothesized that a certain degree of oxidative stress was necessary to induce autophagy, but intensive stress might somehow inhibit autophagy or pyroptosis.

iNOS (inducible nitric oxide synthase) is a prominent enzyme to be responsible for the production of ROS and nitric oxide (NO) during a variety of pathophysiological processes (Anavi & Tirosh, 2020). There was an obvious upregulation of iNOS level in the acrylamide‐treated group. Interestingly, the increase of iNOS was not accompanied by the accumulation of NO. Nitric oxide was active and was involved in a wide range of processes such as the modulation of immune response and regulation of cell apoptosis. In our results, NO secretion was inhibited, which coincided with the results of phagocytic activity. The decreased level of NO was reasonable, which was a cue that the defensive response of cells to environmental stresses was impaired. What's more, it is also reported that NO functions as a bifunctional regulator in cell survival (Picón‐Pagès et al., 2019; Rana, 2020). A persistently high level of nitric oxide (NO) is responsible for mediating inflammation and cellular injury (Sagar et al., 2017). However, exogenous NO also plays an anti‐apoptotic role in hepatocytes. NO is also a critical immune signal in macrophages, besides its role in signal transduction in regular cells. In our study, acrylamide damaged the immune function of macrophages, so nitric oxide (NO) secretion decreased. As iNOS plays a part in the production of both ROS and NO, it is possible that iNOS might largely contribute to the production of ROS rather than NO when cells were exposed to a lower concentration of acrylamide. In addition, the expression of iNOS was inhibited by the P2X7 antagonist. Interestingly, the expression of iNOS was neither like the changes of cell autophagy and pyroptosis nor like the changes of apoptosis, implying the complex role of iNOS in acrylamide‐induced cell death. Further research was required to elucidate this complexity.

In this study, autophagy and apoptosis were excessively activated in RAW 264.7 cells. However, it is reported that normal autophagy was inhibited in U2OS cells (human osteosarcoma cells) after acrylamide treatment for 6 and 24 hs (Song et al., 2021), although the apoptosis rate increased. It seems that the toxic mechanism of acrylamide‐induced PCD is cell‐specific. Moreover, the relationships between different types of PCD need further elucidation.

Yam polysaccharides have been reported to improve the phagocytic function of macrophages. This study further found that it also effectively inhibited acrylamide‐induced programmed cell death, including apoptosis, autophagy, and pyroptosis. We still noted that YPS alone, to some degree, affected the normal level of LC3 and cleaved caspase‐3, but it seems that the levels of autophagy and apoptosis were close to those of the control group. Particularly, the level of TNF‐α somehow increased in YPS‐alone treatment group compared to the control group, which was consistent with the previous report (Li et al., 2017). TNF‐α is related to cell death associated with the immune activity of macrophages, so it is not easy to evaluate the complex changes of TNF‐α. However, it is worth noting that YPS alone promoted the secretion of TNF‐α, but it can effectively control the level of TNF‐α to restore it to the normal level when cells are damaged by acrylamide.

Yam polysaccharides (YPS) upregulated the expression of P2X7 rather than restrain its protein expression. However, during acrylamide‐induced injury, YPS did not further worsen acrylamide‐induced activation of P2X7, although it alone promoted the level of P2X7. It is speculated that the protective effect of Yam polysaccharides may be through other effective targets such as Toll‐like receptor 4 (TLR4) (Li et al., 2017) or more complex signaling pathways, although acrylamide induced programmed cell death (PCD) due to P2X7 stimulation, based on the present results. These possibilities will be explored in our further research.

In addition, Yam polysaccharides have good antioxidant activity, but only prevented the expression of iNOS. Yam polysaccharides did not restore the level of HO‐1 during oxidative damage when a high concentration of acrylamide was applied. Surprisingly, the expression of HO‐1 in the co‐treated group was even slightly lower than that in the acrylamide group. We cannot exactly explain this phenomenon presently, but we will explore the reasons in further research.

**FIGURE 11:**
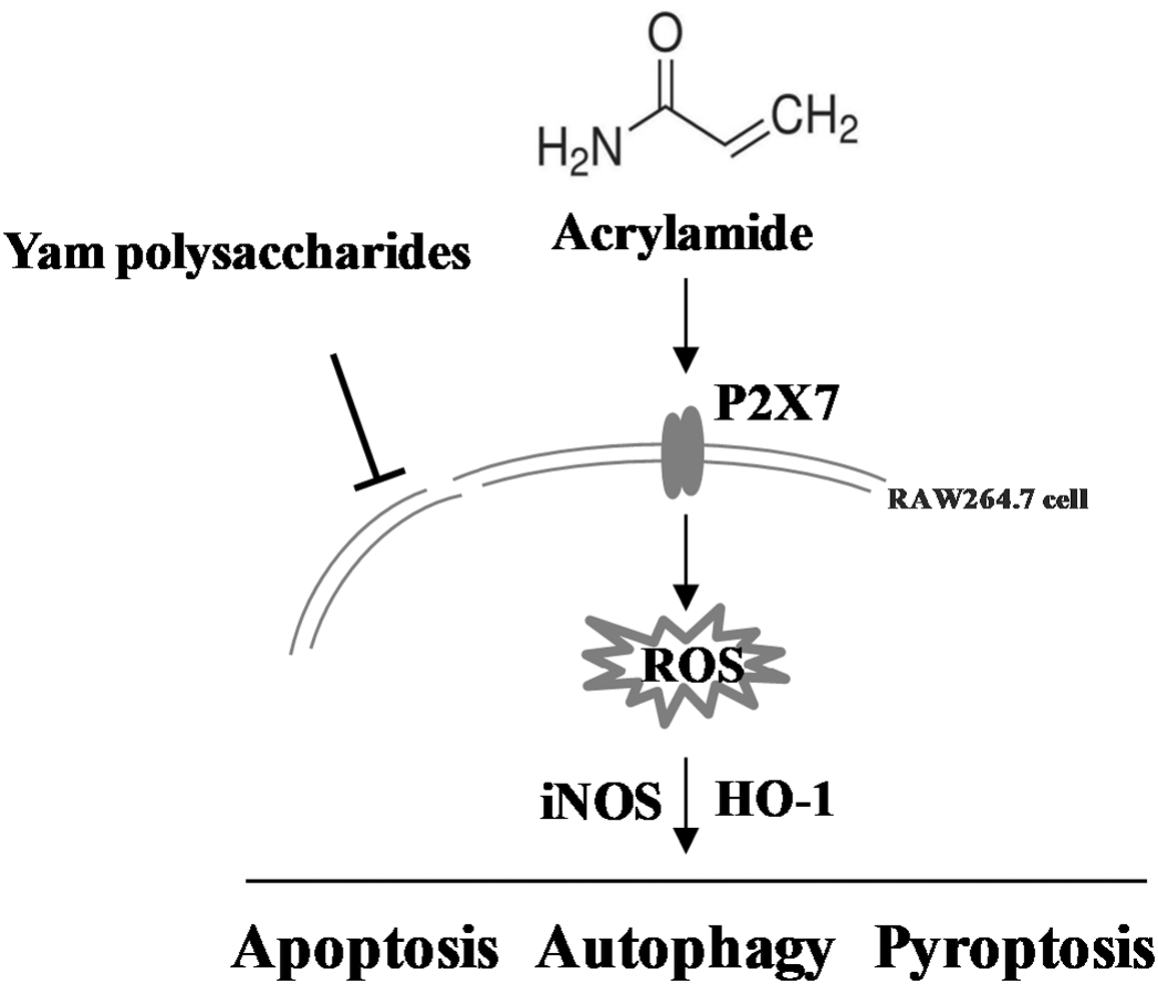
Schematic of the proposed mechanism for the toxic effect of acrylamide and protective effect of Yam polysaccharides (YPS) on RAW 264.7 cell (see text for details). → indicates activation and ⊥ indicates inhibition

Taken together, as proposed in Figure 11, it is demonstrated that acrylamide caused cytotoxicity in RAW 264.7 cells via oxidative stress. Lower concentration of acrylamide led to cell death due to pro‐apoptosis as well as excessive autophagy and pyroptosis. However, apoptosis played the main part in cell death when a higher concentration of acrylamide was used. P2X7 played a vital role in acrylamide‐caused cell death, mainly through regulation of the process of autophagy and pyroptosis. Yam polysaccharides could effectively prevent acrylamide‐induced programmed death in macrophages while an inhibitory effect toward P2X7 was not observed. These findings provide new evidence for clarifying the immunotoxicity of acrylamide and the prevention of its hazard.

## CONFLICT OF INTEREST

The authors declare that there is no conflict of interest.
